# Representations of personalised medicine in family medicine: a qualitative analysis

**DOI:** 10.1186/s12875-022-01650-w

**Published:** 2022-03-01

**Authors:** Marie S. Boyer, Daniel Widmer, Christine Cohidon, Béatrice Desvergne, Jacques Cornuz, Idris Guessous, Daniela Cerqui

**Affiliations:** 1 Department of Family Medicine, Centre for Primary Care and Public Health (Unisanté), Lausanne, Switzerland; 2 General Practitioner in private practice, Lausanne, Switzerland; 3 Centre for Integrative Genomics, National Centre of Competence in Research Frontiers in Genetics, University of Lausanne, Lausanne, Switzerland; 4 Department of Community Medicine, Primary Care and Emergency Medicine, Geneva University Hospitals, Geneva, Switzerland; 5 Institute of Social Sciences, Faculty of Social and Political Sciences, University of Lausanne, Lausanne, Switzerland

**Keywords:** Personalised medicine, Family medicine, Conceptual confusion, Representation, Definition, Health concept

## Abstract

**Background:**

The promise of personalised medicine (PM) to transform healthcare has sparked great enthusiasm in the last years. Yet, its lack of consensus around the nature and scope of the concept has ended in terminological confusion amongst the users in primary care. We aimed to investigate the perceptions of doctors and their patients in response to this evolving concept. This present article focuses on the general understanding of personalised medicine, underlining the confusion over the concept.

**Methods:**

Semi-structured comprehensive interviews were conducted with 10 general practitioners (GPs) and 10 of their patients. The purposive sampling took into account the doctor’s age, sex, and place of practice (rural/urban); each doctor recruited one patient of the same age and sex. Each interview began with the same open-ended question about the participant’s knowledge of the topic, after which a working definition was provided to continue the discussion. Using the grounded theory method, the analysis consisted of open coding, axial coding and selective coding.

**Results:**

From our present analysis focusing on the general understanding of PM, three main themes representing the concept emerged. The first two representations being “centred on the person as a whole” and “focused on alternative and complementary methods”, in which the therapeutic relationship was stated as key. The third theme “medicine open to innovation” involved the few participants who had a good understanding of the concept and could associate personalised medicine with genomics. For those who value therapeutic relationship, the risks of accepting innovation could result in “fast-food” medicine and interpersonal barriers.

**Discussion:**

PM is predominantly unfamiliar in family medicine. It is misinterpreted as a holistic or integrative type of medicine. This semantic confusion probably lies in the choice of the label “personalised” or from the lack of a uniform definition for the term.

## Background

The concept of personalised medicine has generated great enthusiasm in the last years, promising to revolutionise healthcare with optimised diagnostic, therapeutic and preventive strategies based on an individual’s genomic, epigenomic and proteomic profile [1]. Today, PM is mostly applied in oncology for molecular diagnostic purposes and pharmacogenomics, which allow the prediction response of a specific pharmacological treatment [2–5]. However, considering the prevalence of chronic diseases encountered in family medicine, such as cardiovascular diseases, type 2 diabetes mellitus and neurodegenerative diseases, the PM approach could help improve patient care and disease management with the right tools that target high-risk individuals [6–10]. Scientists in favour of this approach agree that the implementation of PM by general practitioners would focus on active surveillance, encourage patient participation in their care, considerably improve quality of life, and potentially reduce health costs [1]. Even if the concept of personalised medicine is well defined in specialised medical literature, it lacks credibility in general practice in the absence of evidence-based research in this domain.

Consequently, there seems to be a general unawareness and lack of experience of personalised medicine among GPs, which makes its application in family medicine quite challenging [11–19]. Barriers to integrating personalised medicine in clinical practice have been widely discussed in literature [20]. Some of these include a deficiency in basic genetic knowledge, skills for decision-making in daily practice, unfamiliarity with genetic tests available on the market such as direct-to-consumer genetic tests (DTCGT) and limited access to genomic medicine expertise [11–18, 20]*.* It has also been demonstrated that training, education and the development of best practices and guidelines would be key before adopting PM in everyday clinical practice [19].

However, the discourse on the risks and limits of PM is complicated by its terminological ambiguity. The absence of one uniform definition has fostered conflicting interpretations among different professions such as biomedical sciences, pharmaceutical industry and healthcare workers [14]. Some have argued that the definition of PM should include focus on the person, the relationship between carer and patient, and also take into consideration the physical-psychological equilibrium, fearing that genetic medicine will overlook other aspects of individualization [21, 22]. In short, there are several possible conceptualisations in personalised medicine, which can be classified in 3 groups: that of the scientists/geneticists and researchers; that of general practitioners, and that of patients. We focused on the last two conceptualisations.

In Switzerland, PM has certainly sparked much interest amongst many professions, including sociology, anthropology and ethics. Within the general public, direct-to-consumer genetic tests have also grown in popularity in the last years. Owing to this rising interest in genomics, the Leenaards Foundation launched “SantéPerso” (personalised care) in 2017, as a means to promote research and interdisciplinary discussion around genomics and PM [23]. To our knowledge, studies related to personalised medicine that were conducted in Switzerland did not directly address its value and role in family medicine [24–26]. One of the research projects of the Leenaards Foundation seeks to investigate the role of PM in primary care for the prevention of chronic diseases using DTCGT [23, 27, 28].

The present study explored the understanding and knowledge about personalised medicine amongst GPs and their patients (emic perspective) using an open-ended question “what does PM mean for you?”. Secondly, after giving to the interviewees an expert definition of personalised medicine (etic perspective) we investigated how GPs and patients anticipated the prospective application of PM in the management of chronic diseases by reviewing their expectations, foreseen and predicted impacts on patient care and their needs, similarly to Najafzadeh M et al., who recognized that the concept of PM, as defined by geneticists, was not familiar to GPs [20]. We used our dual medical and anthropological perspective to help us consider the importance of the interviewees’ interpretations (emic perspective) and develop the consequences of such perspectives [29].

## Methods

### Design

We chose a semi-structured comprehensive interview method to confer with a total of 20 participants. All interviews started with an open-ended question to capture the participants’ own insights on personalised medicine before providing them with a working definition, based on an expert’s consensus (etic perspective). The information collected allowed us to create a quantitative questionnaire to patients and a Delphi to GPs, therefore completing the quantitative part of the project [27, 28]. Interviews were led by either a general practitioner trained in qualitative research or an anthropologist, and were driven to encourage open discussions.

### Ethical considerations

As the data transcribed were anonymized and no health data was collected, the ethics review board of the Canton of Vaud, Switzerland authorised the project under a simplified and accelerated procedure (Req-2018-00160). All study procedures were conducted in accordance with the SRQR guidelines [30].

### Recruitment

For this study, we solicited GPs in Romandy, the French speaking part of western Switzerland. We used purposive sampling technique and selected GPs based on age, sex and place of practice (rural/urban). Each GP included in the study was asked to each recruit one patient in their practice, of the same age and sex. Altogether, we interviewed 10 general practitioners and 10 patients. An information sheet explaining the purpose of the project was sent to the GP accepting the interview and the latter was responsible to give another explanation sheet to the patient he/she selected. The patient signed the informed consent.

### Data collection

Data collection involved audio recordings of the individual interviews that followed a semi-structured guide. The guide consisted of broad themes, including the general understanding of personalised medicine, its impacts in clinical practice, perceived challenges to integrating PM in family medicine. Each interview began with the same open-ended question about the understanding of the term “personalised medicine”. The discussion then followed the broad headings of the guide with specific probes or examples given if the participant did not bring up the topics spontaneously.

### Analysis

Interview recordings were transcribed verbatim, anonymized and analysed as per techniques used in the grounded theory method [31]. The verbatim transcripts were subsequently coded (a GP and an anthropologist coded the first 10 interviews, and all 20 interviews were again coded by 2 GPs), using the qualitative data analysis software MAXQDA12, creating a coding manual after the first 10 interviews. After coding all 20 transcripts, no new salient items emerged, indicating that we reached saturation in salience [32]. Coders exchanged and discussed the interpretation of data patterns and used a constant comparison method. Axial coding followed, in order to release pairs of properties, and finally for selective coding, team members reached a common ground through several discussions. This process of triangulation for data analysis enhanced reliability of the process and the results [33].

The data were separated to present a first set of results with special focus on the participants’ personal perception of PM (emic), before being given a working definition. A second article will be published at a later date, where we will address the participants’ responses after we introduced the concept to them. Quoted statements from the candidates were translated verbatim to English as much as plausible by one of the English-speaking co-authors, to support our data.

## Results

In total, twenty participants were interviewed: 10 general practitioners and 10 patients. 8 participants lived/practised in the rural areas of Romandy, and 12 participants were from the urban region. The age range varied between 35 and 70 years old, and 8 of the participants were female (4 GPs and 4 patients).

**Fig. 1:**
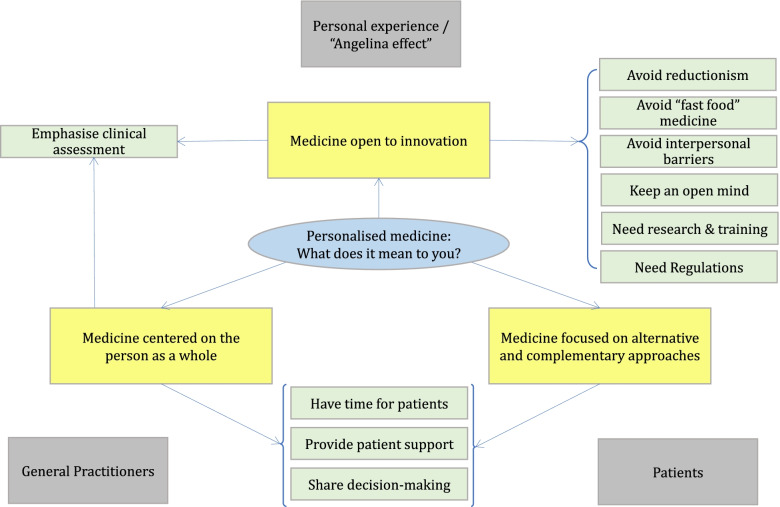
Concept map showing the knowledge of personalised medicine from the participants’ points of view

Overall, despite the information sheet provided, the majority of participants had limited knowledge and little to no experience with personalised medicine. From the analysis, three main representations of the personalised medicine emerged and data from the participants’ perspective were further evaluated and were organised into a concept map (Fig. 1). Quoted statements have been included in order to support this map.

### Medicine centred on the person as a whole

Most GPs interviewed interpreted PM as an approach that considers the physical, psychological and social characteristics of the person. They agreed that the term “personalised” means to nurture interpersonal relationships, communication and sharing of information and trust. GPs mention that having enough time for consultations and providing patient support and treatment guidance are fundamental in clinical practice.

Through this approach, they want to have a global vision of each patient’s situation:

*“It is about recognising the patient as unique, beyond just his genetics and heredity. It’s about considering his psychological functioning, life experience, ability to manage stress, profession, social status and then the medical conditions or risks thereof. It’s a whole package!” (M4)*

In Switzerland, GPs are often under time constraints, considering that fees are based on time and time is restricted. However, GPs want to be more involved in patient care through the possibility of having enough time for consultations, patient support and counselling:

*“We already apply personalised medicine. In general medicine, we certainly already use it, without being in the specifics of genetic tests and the study of the genome. In my opinion, I don’t know if others do … but I define this (my profession) as personalised medicine there’s involvement and sharing of information with the patient* [ … ]*” (M9)*

*“I hope to have always the time to discuss, to be able to give one hour to a patient who has existential or life questions.* [ … ] *Doctors are always needed to provide support” (M2)*

Their patients expressed a similar understanding of PM, where the person is the focal point. They emphasised the doctor-patient relationship, where GPs would take the time to listen and involve them in all aspects of care:

*“Nurses and doctors are now increasingly involved in patient care, and show concern for the patient. We now find an approach focused on the person in patient care and management, care that is centred on his needs, on his characteristics* [ …]. *” (P7).*

### Medicine focused on alternative and complementary approaches

Some patients referred to non-conventional approaches of medicine, where alternative and complementary methods meet traditional evidence-based medicine. When asked to give examples of personalised medicine, a patient brought up their naturopathic practitioner, who regards the person on both the physical and psychological levels:

*“I see a naturopathic practitioner, and I think she does that. Because she enquires about my emotions, my symptoms, and yes, she takes a global approach to my being. It’s about more than just telling me « you have a pimple, let’s see … does it itch? Etc etc* [ … ]*»” (P3)*

This interpretation of personalised medicine also derives from the caring perspective. It shares similarities with that of “the person as a whole”, in the way that one keeps the overall vision in mind, take the time for patients and provide them with the support required. According to the participants, these are prerequisites before introducing any medical innovations.

### Medicine open to innovation

This representation encompasses genomics and other new technological advancement of medicine. A few participants provided a relatively accurate definition of PM:

*“So for me, what first comes to mind … is mainly research on a genetic level, to study either diseases, or specific genes which could be used to develop treatment for a patient* [ … ]*” (M10)*

*“A type of medicine that is customised … truly individualised for each patient … based on their genetic characteristics. As you see, my body, my heredity is - umm - different from another person. So, I probably have (genetic) mutations … An effective treatment for me, is not necessarily so for someone else … ” (P7)*

These interviewees had knowledge of personalised medicine either from personal experience (in the area of oncology) or from the media. PM had indeed stimulated much public interest and awareness in 2013, from the widely broadcasted “Angelina effect”, in connection to heredity breast cancer.

*“*[ … ] *I read in the newspaper that Angelina Jolie had a double mastectomy because she did a genetic test, which showed that she had a high probability to develop breast cancer.” (P10)*

Despite the perceived benefits on a therapeutic level, GPs insisted that the emphasis of patient care should remain on clinical assessment.

*“I would still tend to say: “let’s do a physical exam (e.g. a cardiac exam) to have a baseline”. Then, I would advise my patients to have a follow-up a couple of years later in case. Even if the genetic risk profile is negative.” (M3)*

As a consequence of PM, a few doctors believe they would be relying more on standardised algorithms in patient management, thereby assuming a reductionist approach that could potentially alter interpersonal relationships. One patient expressed similar opinions and compared genomics to “fast food” medicine.

*“Because we rely on many standards and values such as, I don’t know, laboratory values, imaging tests, or other. And patients are also very … I want to say a little too dependent on these lab results, scan report. And when we are too absorbed in something, we lose the overall picture. So, I believe that it will somehow put a screen between doctors and the patient.” (M1)*

*“I think the relationship between doctor and patient is multi-layered, so to say. There’s also social communication, which is as important, if not more [important], than medications. So, if you remove this layer and respond with “fast food” that is based on algorithms, this social role disappears. And from my experience on life and human relationships, without this, medicine would lose much of its usefulness.” (P5)*

Many dread the advent of interpersonal barriers from the implementation of PM in clinical practice.

*“I think that our society will go a little adrift with all this. In my opinion, we lose our true bearings and what’s important in life, relationships between people, empathy, fondness, affection and happiness. … I find that people are already quite self-absorbed and all about keeping focus on oneself, and I find this quite deleterious.” (M4)*

With the right tools at hand, some GPs will be willing to implement PM in family medicine.

*“It’s inevitable. Because techniques move forward, so we cannot go against the change. I think we should just go with the flow.” (M2)*

Last but not least, our interlocutors have highlighted uncertainties and challenges around the integration of genomic medicine in primary care. Of those, the need for research and further education to adapt to the change and manage possibly high demands, has emerged:

*“It would be necessary for doctors to be trained to know how to communicate the information efficiently and appropriately, and also to manage the stress that it (genetic information) can generate for the patient* [ …]. *” (M1)*

Furthermore, they expressed the necessity of regulatory measures facing innovation, even if this does not seem easy.

*“So, I am for PM to be state regulated* [ … ] *I support freedom of choice, but while keeping solidarity and community spirit in mind. There are many issues with the present society, which is one with selfish people, one that is increasingly focused on personal gain.” (M8)*

*“The problem is that there are two components of the law, there’s justice and justness. And in between, there’s all the issues concerning humans, lobbies, influences and ignorance.” (P5)*

## Discussion

Our study highlights that personalised medicine is a well-established concept in medical literature but it bears an entirely different meaning in primary care. From our results, it outlines a more integrative or holistic approach. In other terms, participants have largely confused PM with person-centred medicine or even integrative medicine.

In primary care, the key point is to devote support and time to the patients for the shared decision-making process. For the few participants who understood that we were talking about medicine open to innovation, it was also important not to forget the doctor-patient relationship by promoting a reductionist vision. Innovation could only be introduced through research, training and regulation.

The therapeutic relationship between carer and patient has evolved over the years, from the paternalistic model towards patient autonomy. One of the fundamental characteristics of family medicine, as defined by the WONCA, the academic association for general practice, is to develop a person-centred approach [34]. The idea that a person is more comprehensive than a patient finds its roots in the tradition of general medicine [35]. El-Alti et al. have previously stated that person-centred care and PM can share some similarities, in that both seek to shift from the “standardised, one treatment fits all” model [21]. They outlined person-centred medicine as an approach that is holistic, non-reductionist and deriving from a caring perspective, where the carer and the patient shares a relationship based on individuality and sharing [21]. Vogt et al., who attempted to find the link between integrative concept of biopsychosocial medicine and systems biology, concluded that the latter concept could not be viewed as person-centred in a humanistic model of medicine [36].

A primary cause for misinterpretation probably lies in the choice of the label “personalised” to define a technology that is derived from genomics. It could be speculated that renaming this technology with a more precise term could be less ambiguous. It should be noted that there is a non-exhaustive list of terms that have been used interchangeably in literature over the years, including precision medicine, genomic medicine, stratified medicine and individualized healthcare [37]. Besides terminological ambiguity, the lack of consistency in its definition and the scope of the concept have also been largely disputed in literature [14, 20, 37].

Schleidgen et al. proposed a more precise definition of personalised medicine in their paper, in an attempt to help clarify the conceptual differences between stakeholders concerned with PM [14]. According to the authors, a shared understanding of the concept could facilitate the discourse on the nature, the risks and the limits of PM [14]. However, De Grandis and Halgunset largely scrutinized their work and believed that scientists should remain open to the vagueness of the concept of PM, as it is still an “ideal” with varying views and visions from different stakeholders [37]. Other terminological confusions encountered in general medicine is the notion of P4 medicine, which is defined as predictive, preventive, personalised and participatory care [38] and the concept of quaternary prevention, concerned by the risk of over-medicalisation, that has also been named P4 in general practice [39]. This confusion is quite troubling, given the remarks in published literature on the risk of over-medicalisation from the advent of new technologies, particularly of personalised medicine [40].

### Strengths and limitations

The main strength of this study is the inclusion of the perspective of both patients and GPs, giving a global representation of family medicine in both rural and urban regions of the French-speaking part of Switzerland. Furthermore, the team involved in this study is multi-disciplinary, with GPs, a public health doctor, a geneticist and an anthropologist. This ensured that the topics covered in the interviews involved the issues considered by key stakeholders.

Collaborating with an anthropologist also led us to adopt an emic (interviewee’s point of view) rather than an etic (researcher’s point of view) approach.

The purposive sampling of this study could be disputed as participating GPs chose the patients, based solely on criteria of the same age and sex. None of the researchers were involved in the recruitment of patients. Participating GPs have often admitted that patient recruitment for such a topic was no easy task. So, it is possible that the selection of patients was conditioned by some prior knowledge or interest. It is indeed difficult in such research to start from knowledge devoid of any background information. Another limitation is the sample size, although small, was large enough so that no new salient themes emerged after coding all twenty interviews. Therefore, it could be argued that the data are not necessarily generalizable to all GPs and users in family medicine. We must remember that this qualitative study was the preliminary part of a bigger one.

All participants were interviewed on a voluntary basis and were all given an information sheet, briefly explaining the study. Both groups were informed that personalised medicine involved genetic risk profiling. However, details such as the aim and methodology of the study were additionally given to GPs. Although GPs had a more in-depth overview of the study, interviews were conducted with the same open-ended question and were led as an open discussion.

## Conclusion

The present study emphasises the need to reach consensus on the definition of personalised medicine, while taking in consideration all key stakeholders’ objectives and interest. A prerequisite to the implementation of genetic medicine in family practice is to understand the different points of view and to take into account the different languages: molecular biology and socio-cultural ones [41].

In order to avoid any terminological ambiguity, we believe that “Genomic medicine” would be an appropriate term, considering the necessity of using genetic material to exploit this technology. However, according to Jaccard et al., “precision medicine” would be more fitting to limit the widespread opinions amongst GPs that medical care is already “personalised” [42]. Precision medicine also includes big data and connected devices in addition to genomics: a concept that does not seem as clear to patients.

In this study, GPs would clearly have the pivoting role in interpreting genetic information for the patients in a shared-decision process. Hence, doctors stressed the need for further research, training and education, including guidance in the interpretation of genetic risk profiles, so as to limit the risks of over-medicalisation [41].

## Data Availability

The data used and/or analysed during this study are available from the corresponding author on reasonable request.

## Abbreviations

PM: Personalised medicine
GP: General Practitioner
DTCGT: Direct-to-consumer genetic test
SRQR: Standards for Reporting Qualitative Research
